# Factors Associated With Walking Speed in Older Adults With and Without Sarcopenia: A Cross-Sectional Study in Long-Term Care Facilities

**DOI:** 10.7759/cureus.83219

**Published:** 2025-04-29

**Authors:** Takumi Jiroumaru, Yutaro Hyodo, Kenji Mori, Tomoka Hattori, Ikkei Tanaka, Yasumasa Oka, Junko Ochi, Nobuko Shichiri, Takamitsu Fujikawa

**Affiliations:** 1 Physical Therapy, Bukkyo University, Kyoto, JPN; 2 Rehabilitation, Kanazawa Orthopaedic and Sports Medicine Clinic, Ritto, JPN; 3 Occupational Therapy, Bukkyo University, Kyoto, JPN

**Keywords:** dynamic balance, handgrip strength, long-term care, older adults, physical function, respiratory muscle strength, sarcopenia, two-step test, walking speed

## Abstract

Background: Sarcopenia is characterized by the progressive loss of muscle mass and function and is associated with an increased risk of fractures, disability, and mortality. Previous studies have explored the relationship between sarcopenia and physical function in community-dwelling older adults; however, limited research has focused on individuals requiring support or long-term care in populations where the effect of sarcopenia may be more severe. Therefore, the aim of this study was to investigate the factors associated with walking speed in older adults with and without sarcopenia living in long-term care facilities.

Methods: This cross-sectional study included 47 older adults (aged ≥ 65 years) who attended daycare facilities in Shiga, Japan. Participants were categorized into sarcopenia and non-sarcopenia groups based on the 2019 criteria of the Asian Working Group for Sarcopenia (AWGS). Physical function was assessed using respiratory muscle strength (maximum inspiratory pressure (PImax) and maximum expiratory pressure (PEmax)), grip strength, walking speed (six-meter walk test), and dynamic balance (maximum distance in the two-step test (MDST)). Pearson’s correlation coefficient and stepwise multiple regression analyses were used to examine the association between walking speed and physical functional parameters.

Results: Walking speed significantly correlated with PEmax (r = 0.569, p = 0.002), grip strength (r = 0.500, p = 0.008), and MDST (r = 0.716, p < 0.001) in the non-sarcopenia group. PEmax (β = 0.387, p = 0.017) and MDST (β = 0.600, p < 0.001) were significant predictors of walking speed, explaining 60.6% of its variance (adjusted R² = 0.606). However, no significant associations or predictive variables were observed in the sarcopenia group, likely because of advanced muscle deterioration and confounding factors, such as comorbidities.

Conclusions: Walking speed in older adults without sarcopenia was significantly associated with respiratory muscle strength and dynamic balance, suggesting that targeted interventions such as respiratory muscle training and balance exercises may help maintain or enhance mobility. Older adults with sarcopenia may require comprehensive interventions, including nutritional support and generalized muscle strengthening.

## Introduction

Sarcopenia is a geriatric syndrome characterized by a progressive decline in muscle mass and function, associated with an increased risk of fractures, functional deterioration, and mortality [1]. With a global prevalence estimated at 10-16% among older adults [2], sarcopenia represents a major public health concern. In Japan, its prevalence among community-dwelling older adults is 11.5% in men and 16.7% in women [3], with even higher rates observed among individuals in nursing homes, likely owing to their more advanced age and greater frailty [4]. Sarcopenia has been associated with alterations in biological markers related to inflammation [5], metabolism [6-8], and vascular function [9]. These findings indicate that sarcopenia affects multiple physiological systems, although the exact mechanisms remain unclear [5-9].

Walking speed is a simple yet powerful predictor of health outcomes and mortality in older adults and is often referred to as the “sixth vital sign” [10]. Several physical functions influencing walking speed have been identified, including respiratory muscle strength [11], handgrip strength [12], and dynamic balance [13]. Respiratory muscle strength reflects the overall muscular condition, and its decline may contribute to generalized functional impairment [14]. Similarly, reductions in dynamic balance increase the risk of falls and fractures [15]. Handgrip strength is widely used as a proxy for overall muscle strength and is considered a reliable predictor of functional status and health [16].

Although numerous studies have investigated the relationship between sarcopenia and physical function in community-dwelling older adults, research remains limited among individuals requiring support or long-term care [17,18]. This population often experiences a pronounced decline in physical ability, amplifying the functional consequences of sarcopenia [18]. Previous research has suggested associations among respiratory muscle strength, dynamic balance, and walking speed in this group, highlighting the need for comprehensive physical assessments [19]. However, the extent to which these physical functions contribute to walking speed based on sarcopenia status remains unclear.

Additionally, few studies have investigated whether the relative effect of specific functional components, such as grip strength or balance, on walking speed differs between individuals with and without sarcopenia [17,19]. Clarifying these relationships could determine whether standard assessment tools such as grip strength and walking speed are sufficiently sensitive for detecting functional changes in frail older populations, particularly those requiring long-term care.

This study aimed to identify the factors associated with walking speed in older adults with and without sarcopenia living in long-term care facilities. Specifically, the objectives were: (i) to classify participants into sarcopenia and non-sarcopenia groups; (ii) to assess key physical functions, including respiratory muscle strength (maximum inspiratory pressure (PImax) and maximum expiratory pressure (PEmax)), handgrip strength, and dynamic balance (two-step test); and (iii) to determine the physical factors most strongly associated with walking speed in each group. The findings are expected to inform the development of tailored intervention strategies to maintain or enhance mobility in frail older populations.

## Materials and methods

Study design

This cross-sectional study was conducted between December 2023 and May 2024 at daycare centers located in a mid-sized city in Shiga, Japan. All participants received verbal and written explanations of the study and provided written informed consent before participation. The study was conducted in accordance with the principles of the Declaration of Helsinki and was approved by the Ethics Committee of Kanazawa Orthopaedic and Sports Medicine Clinic (approval number: Kanazawa-OSMC-2023-003).

Participants

In total, 47 community-dwelling older adults (16 men and 31 women) aged 65 years or older who were using daycare services in Japan were recruited. The age criterion of ≥ 65 years was chosen according to the World Health Organization’s definition of older adults and because this group typically qualifies for Japan’s long-term care insurance system. The sample size was determined based on the number of available and eligible individuals attending the collaborating daycare facilities during the recruitment period, reflecting a pragmatic and feasible approach to participant enrollment for an exploratory cross-sectional study. All the participants were certified as requiring assistance or long-term care under the Japanese long-term care insurance system. The participating facilities offered rehabilitation programs, including exercise sessions and transportation services. None of the participants had any cognitive impairment. Participants were either able to walk independently or used walking aids as part of their daily living. Individuals with severe pain or postural abnormalities that limited respiratory muscle strength, handgrip strength, or walking speed testing were excluded.

**Figure 1:**
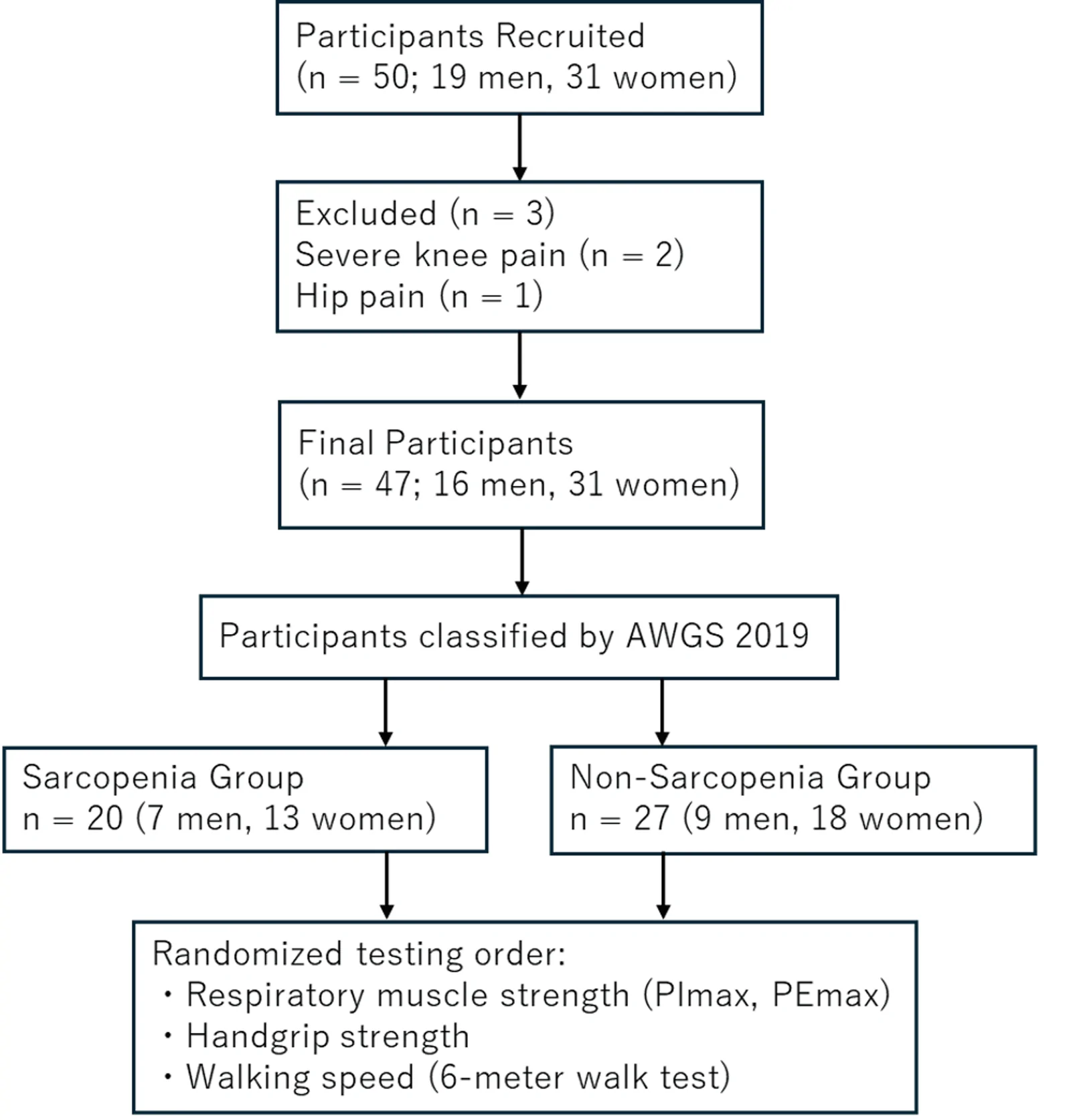
Flowchart of participant recruitment, classification, and group allocation AWGS: Asian Working Group for Sarcopenia; PEmax: Maximum expiratory pressure; PImax: Maximum inspiratory pressure

Figure 1 presents a flowchart of the recruitment process and grouping of study participants. Although 50 participants (19 men and 31 women) were initially recruited, three patients had to be excluded because of pain that substantially impaired walking (severe knee pain, n = 2; hip pain, n = 1). Thus, the final cohort comprised 47 participants (16 men and 31 women). The participants were divided into sarcopenia (n = 20; seven men and 13 women) and non-sarcopenia groups (n = 27; nine men and 18 women) based on the Asian Working Group for Sarcopenia (AWGS) 2019 algorithm [20]. The order of the physical function tests (respiratory muscle strength, handgrip strength, and walking speed) was randomized to avoid order bias.

Respiratory muscle strength

Respiratory muscle strength was assessed using PImax and PEmax. A spirometer (Autospiro AS-507, Minato, Japan) was used for all measurements, which a trained physical therapist conducted in accordance with the American Thoracic Society/European Respiratory Society guidelines [21]. The highest values of the two trials for PImax and PEmax were recorded and used for analysis.

Handgrip force

Handgrip strength was measured using a Smedley dynamometer (TKK5401 Grip-D, Takei Scientific Instruments, Japan). The measurements were performed twice for each hand while the participants were seated. The highest value obtained across trials was used for the analysis.

Walking speed

Walking speed was measured using a six-meter walk test on a flat indoor surface. The test procedure followed the standardized protocol recommended by the AWGS 2019 guidelines [20]. A physical therapist supervised all the trials to ensure safety. The participants practiced the test 3-5 times beforehand and wore regular footwear. They were instructed to begin walking from a stationary standing position and continue at their usual pace without deceleration near the six-meter mark. The time required for the lead foot to travel from the start line to the six-meter line was measured using a handheld stopwatch. The average walking speed (m/s) was calculated by dividing distance by time. The test was performed twice, and the faster of the two trials was used in the analysis. Participants were allowed to use personal mobility aids (e.g., canes or walkers); however, physical assistance was not allowed.

Dynamic balance

Dynamic balance was assessed using the maximum two-step test (MDST), which was supervised by the same physical therapist who conducted the walking speed assessments. The test procedure is a standardized method for evaluating locomotive syndrome, a condition characterized by impaired mobility due to musculoskeletal disorders, as described in a previous study [19]. The test was conducted on a flat surface within daycare centers. The participants performed two test trials in a standing position after practicing the procedure 3-5 times while wearing their usual footwear. The MDST measured the maximum distance (cm) between the toe of the starting foot and the heel of the second foot. The order of the stepping foot was not standardized. Each MDST value was divided by the participant’s height (cm) to adjust for body size. All the tests were conducted under strict supervision to prevent falls. No physical assistance was provided, although personal assistance devices were permitted.

Skeletal muscle mass

Skeletal muscle mass was measured using a multifrequency bioelectrical impedance analysis (BIA) device (InBody 520, InBody, Japan). The skeletal muscle mass index (SMI) was calculated by dividing the sum of the appendicular skeletal muscle mass (kg) by the square of height (m²), following the standardized method recommended by the AWGS 2019 guidelines [20].

Sarcopenia assessment

Sarcopenia was diagnosed based on the AWGS 2019 criteria, which include reduced muscle mass, muscle strength, and physical performance. The diagnostic cut-off values were as follows: handgrip strength < 28.0 kg for men and < 18.0 kg for women, walking speed < 1.0 m/s for both sexes, and SMI < 7.0 kg/m² for men and < 5.7 kg/m² for women [20].

Statistical analysis

Associations between walking speed and physical function variables (PImax, PEmax, handgrip strength, and MDST) were examined using Pearson’s correlation coefficients and multiple regression analysis within the sarcopenia and non-sarcopenia groups. Before analysis, the distribution of all variables was tested for normality using the Shapiro-Wilk test, confirming a normal distribution.

A stepwise multiple regression analysis was conducted with walking speed as the dependent variable and PImax, PEmax, handgrip strength, and MDST as independent variables, with adjustments for sex. Multicollinearity was assessed using the variance inflation factor (VIF) to ensure that all the values were within acceptable limits. Statistical analyses were performed using IBM SPSS Statistics, version 27 (IBM Corp., Armonk, USA), with a significance threshold set at p < 0.05.

## Results

Before conducting data analyses, the normality of all quantitative variables was confirmed using the Shapiro-Wilk test, indicating normal distributions in the sarcopenia and non-sarcopenia groups. Table 1 presents the descriptive characteristics of the participants stratified according to sarcopenia status. The categorical variable “educational background” was dummy-coded for inclusion in the regression models. The correlation matrices indicated no variables with an absolute correlation coefficient (|r|) > 0.8 in either group, allowing for the inclusion of all variables in subsequent analyses. Multicollinearity was also assessed using the VIF, and all were below the threshold of 10.0. The Durbin-Watson statistic values were 1.869 and 2.444 for the sarcopenia and non-sarcopenia groups, respectively, suggesting no significant autocorrelation of the residuals. No outliers exceeding ± 3 standard deviations from the predicted values were detected in either group.

**Table 1:** Characteristics of participants according to group (sarcopenia vs. non-sarcopenia) Values are presented as mean ± standard deviation. BMI: Body mass index; SMI: Skeletal muscle mass index; PEmax: Maximum expiratory pressure; PImax: Maximum inspiratory pressure; MDST: Maximum distance in the two-step test

Variable	Total (n = 47)	Sarcopenia group (n = 20)	Non-sarcopenia group (n = 27)
Age (years)	82.91 ± 5.99	83.38 ± 2.56	82.58 ± 7.46
Sex (men/women)	16/31	7/13	9/18
BMI (kg/m²)	22.91 ± 3.09	21.40 ± 2.53	24.41 ± 2.85
SMI (kg/m^2^)	6.27 ± 0.86	5.74 ± 21.40	6.67 ± 0.75
Handgrip strength (kg)	20.99 ± 8.01	19.10 ± 6.15	22.39 ± 8.88
Walking speed (m/s)	0.76 ± 0.25	0.73 ± 0.21	0.77 ± 0.28
PImax (cmH₂O)	37.37 ± 17.90	32.45 ± 6.15	41.02 ± 15.79
PEmax (cmH₂O)	49.47 ± 22.93	40.77 ± 19.93	55.92 ± 22.88
MDST (cm/cm)	0.87 ± 0.23	0.87 ± 0.20	0.87 ± 0.25

Pearson’s correlation analysis revealed no significant relationships between the measured variables in the sarcopenia group. In contrast, the non-sarcopenia group demonstrated several significant positive correlations with walking speed: PImax (r = 0.392, t = 2.145, p = 0.043), PEmax (r = 0.569, t = 3.452, p = 0.002), handgrip strength (r = 0.500, t = 2.933, p = 0.008), and MDST (r = 0.716, t = 5.564, p < 0.001) (Table 2).

**Table 2:** Pearson’s correlation coefficients between walking speed and physical function variables Pearson’s correlation coefficient (r), corresponding t-statistic (t), and p-value (p) were calculated to assess the relationships between walking speed and physical function variables. Degrees of freedom were 18 for the sarcopenia group and 25 for the non-sarcopenia group. * denotes p < 0.05, indicating statistical significance. PEmax: Maximum expiratory pressure; PImax: Maximum inspiratory pressure; MDST: Maximum distance in the two-step test

Variable	Sarcopenia group (n = 20, df = 18)	Non-sarcopenia group (n = 27, df = 25)
PImax (cmH₂O)	r = 0.143, t = 0.615, p = 0.537	r = 0.392, t = 2.145, p = 0.043*
PEmax (cmH₂O)	r = 0.123, t = 0.524, p = 0.600	r = 0.569, t = 3.452, p = 0.002*
Handgrip strength (kg)	r = 0.174, t = 0.749, p = 0.443	r = 0.500, t = 2.933, p = 0.008*
MDST (cm/cm)	r = 0.264, t = 1.152, p = 0.260	r = 0.716, t = 5.564, p < 0.001*

Stepwise multiple regression analysis identified a significant model only in the non-sarcopenia group. This model explained 60.6% of the variance in the walking speed (adjusted R² = 0.606). PEmax and MDST were the significant contributors. Specifically, a one standard deviation increase in PEmax was associated with a 0.387 standard deviation increase in walking speed (SE = 0.002, β = 0.387, p = 0.017). Similarly, a one standard deviation increase in MDST was associated with a 0.600 standard deviation increase in walking speed (SE = 0.139, β = 0.600, p < 0.001). These findings indicate that both expiratory muscle strength and dynamic balance have meaningful and significant effects on walking speed in older adults without sarcopenia (Table 3).

**Table 3:** Stepwise multiple regression analysis for predictors of walking speed in the non-sarcopenia group A stepwise multiple linear regression analysis was performed with walking speed as the dependent variable. * denotes p < 0.05 and ** denotes p < 0.01, indicating significant predictors. The adjusted R² of the model was 0.606, indicating that the predictors explained approximately 60.6% of the variance in walking speed. PEmax: Maximum expiratory pressure; MDST: Maximum distance in the two-step test; SE: Standard error

Predictor	β	SE	t-value	p-value	95% confidence interval	Adjusted R^2^
PEmax (cmH₂O)	0.387	0.002	2.576	0.017*	0.001, 0.008	0.606
MDST (cm/cm)	0.600	0.139	4.634	< 0.001**	0.358, 0.934	

## Discussion

This study classified older adults receiving assistance or long-term care into sarcopenia and non-sarcopenia groups and examined the relationships between walking speed and key physical function indicators, including respiratory muscle strength, handgrip strength, and dynamic balance.

In the non-sarcopenia group, significant correlations were observed between walking speed and respiratory muscle strength (PEmax and PImax), handgrip strength, and dynamic balance. Stepwise multiple regression analysis identified PEmax and MDST as significant predictors of walking speed, suggesting that expiratory muscle strength and dynamic balance play crucial roles in ambulatory function. Respiratory muscle strength reflects trunk muscle function and is associated with postural control and general muscular capacity [22]. These findings are consistent with those of previous studies [19] that reported associations between respiratory muscle strength, dynamic balance, and walking speed in older adults requiring care.

Dynamic balance is a well-established determinant of fall risk in older adults, and interventions aimed at improving balance can enhance gait performance and reduce fall incidence [23]. Balance training, particularly when combined with muscle strengthening, plays a vital role in geriatric rehabilitation [24].

In contrast, the sarcopenia group did not exhibit significant associations between walking speed and the measured physical function variables. This lack of association may be attributed to the multifactorial deterioration in muscle mass, strength, and neural function commonly observed in patients with sarcopenia. Moreover, older adults with sarcopenia are frequently affected by comorbidities and age-related physiological decline, making it challenging to isolate the influence of a single variable on walking ability [25]. Sarcopenia is also associated with chronic inflammation, malnutrition, and systemic diseases [26,27], which collectively impair physical performance.

These results highlight the complexity of sarcopenia and indicate that interventions targeting isolated functional components may be insufficient for this population. Instead, multimodal strategies combining exercise therapy, nutritional supplementation, and medical management should be considered when developing interventions for patients with sarcopenia.

Furthermore, the absence of significant associations in the sarcopenia group may reflect a ceiling effect, wherein physical function declines to a level that minimizes the observable effect of individual factors on walking performance. This finding highlights the importance of early screening and intervention before sarcopenia progresses to a more advanced stage.

A unique strength of this study is its focus on older adults requiring support or care, a population often excluded from research, and the comparison of functional correlates of walking speed between sarcopenic and non-sarcopenic individuals. The divergence in predictive factors between the groups emphasizes the importance of personalized rehabilitation approaches.

For older adults without sarcopenia, targeted interventions such as respiratory muscle training and balance exercises may be beneficial. Respiratory muscle improves exercise tolerance [28], suggesting its use in maintaining mobility and physical function. Similarly, balance training enhances walking ability and reduces fall risk [29], with interventions that address strength and balance concurrently being recommended.

Therefore, more comprehensive approaches are required for older adults with sarcopenia. Nutritional interventions aimed at addressing protein deficiency, combined with resistance training, improve muscle mass and function in frail populations. Tieland et al. [30] demonstrated that protein supplementation enhanced the effects of resistance training in older adults.

Given that older adults receiving long-term care may face challenges in accessing traditional rehabilitation settings, future research should explore the feasibility and effectiveness of home-based or telerehabilitation programs that integrate nutritional support and exercise guidance.

This study has several limitations. First, the overall sample size was relatively small, particularly in the sarcopenia group (n = 20), potentially reducing the statistical power. Although a priori power analysis, based on a large effect size (f² ≈ 1.538) calculated from the adjusted R² value (0.606) in the non-sarcopenia group, indicated that approximately 10 participants were sufficient, the sarcopenia group’s smaller size remains a limitation. Second, the cross-sectional design prevents establishing causal relationships. Third, confounding factors such as nutritional status, cognitive function, psychological health, and comorbidities were not fully controlled. Future studies with larger sample sizes, longitudinal designs, and better adjustment for confounders are needed to validate and strengthen these findings.

## Conclusions

This study compared physical function characteristics related to walking speed between older adults with and without sarcopenia who were receiving assistance or long-term care. In the non-sarcopenia group, respiratory muscle strength (PEmax) and dynamic balance (MDST) emerged as significant predictors of walking speed. In contrast, no significant associations were identified in the sarcopenia group. These findings suggest that for non-sarcopenic individuals, interventions focusing on respiratory muscle and balance training may help maintain or improve mobility. For individuals with sarcopenia, however, more comprehensive approaches like incorporating nutritional enhancement and systemic muscle strengthening are warranted.
