# Correction: Factors Associated With Walking Speed in Older Adults With and Without Sarcopenia: A Cross-Sectional Study in Long-Term Care Facilities

**DOI:** 10.7759/cureus.c475

**Published:** 2026-09-02

**Authors:** Takumi Jiroumaru, Yutaro Hyodo, Kenji Mori, Tomoka Hattori, Ikkei Tanaka, Yasumasa Oka, Junko Ochi, Nobuko Shichiri, Takamitsu Fujikawa

**Affiliations:** 1 Physical Therapy, Bukkyo University, Kyoto, JPN; 2 Rehabilitation, Kanazawa Orthopaedic and Sports Medicine Clinic, Ritto, JPN; 3 Occupational Therapy, Bukkyo University, Kyoto, JPN

Following publication, substantial thematic overlap was identified between this article and a previously published article by the same authors: Hyodo Y, Jiroumaru T, Mori K, et al. Comparison of the effect of respiratory muscle strength on dynamic and static balance assessment between sarcopenia and non-sarcopenia groups. J Phys Ther Sci. 2023;35(10):703–707. DOI: 10.1589/jpts.35.703 Both studies were conducted under the same ethics approval, while the participant datasets, data-collection periods, and outcome measures differed. This notice serves to inform readers and researchers of its relationship to the earlier publication cited above.

